# Research on Water Environment Regulation of Artificial Playground Lake Interconnected Yangtze River

**DOI:** 10.3390/ijerph15102110

**Published:** 2018-09-25

**Authors:** Weiwei Song, Xingqian Fu, Yong Pang, Dahao Song, Qing Xu, Peng Zhang

**Affiliations:** 1 Key Laboratory of Integrated Regulation and Resources Development on Shallow Lakes, Ministry of Education, Hohai University, Nanjing 210098, China; weiweisong0515@163.com; 2 College of Hydrology and Water Resources, Hohai University, Nanjing 210098, China; 3 College of Engineering, University of Miami, Coral Gables, FL 33146, USA; 4 Kewen College, Jiangsu Normal University, Xuzhou 221116, China; 18852196181@163.com (X.F.); 18852196517@163.com (D.S.); 5 College of Environment, Hohai University, Nanjing 210098, China; zhap2014@163.com; 6 School of Hydraulic, Energy and Power Engineering, Yangzhou University, Yangzhou 225009, China; 7 School of Environmental and Municipal Engineering, North China University of Water Resources and Electric Power, Zhengzhou 450045, China

**Keywords:** numerical simulation, eutrophication remediation, Playground Lake, bilateral rivers, unilateral river

## Abstract

With the rapid development of China, water pollution is still a serious problem despite implementation of control measures. Reasonable water environment management measures are very important for improving water quality and controlling eutrophication. In this study, the coupled models of hydrodynamics, water quality, and eutrophication were used to predict artificial Playground Lake water quality in the Zhenjiang, China. Recommended “unilateral” and “bilateral” river numerical models were constructed to simulate the water quality in the Playground Lake without or with water diversion by pump, sluice and push pump. Under “unilateral” and “bilateral” river layouts, total nitrogen and total phosphorus meet the landscape water requirement through water diversion. Tourist season in spring and summer and its suitable temperature result in heavier eutrophication, while winter is lighter. Under pumping condition, water quality and eutrophication of “unilateral” river is better than “bilateral” rivers. Under sluice diversion, the central landscape lake of “unilateral river” is not smooth, and water quality and eutrophication is inferior to the “bilateral”. When the water level exceeds the flood control level (4.1 m), priority 1 is launched to discharge water from the Playground Lake. During operation of playground, when water level is less than the minimum level (3.3 m), priority 2 is turned on for pumping diversion or sluice diversion to Playground Lake. After opening the Yangtze river diversion channel sluice, priority 3 is launched for sluice diversion to the Playground Lake. When the temperature is less than 15 °C, from 15 °C to 25 °C and higher than 25 °C, the water quality can be maintained for 15 days, 10 days and 7 days, respectively. Corresponding to the conditions of different priority levels, reasonable choices of scheduling measures under different conditions to improve the water quality and control eutrophication of the Playground Lake. This article is relevant for the environmental management of the artificial Playground Lake, and similar lakes elsewhere.

## 1. Introduction

Ecological models are important tools for lake eutrophication research and lake ecosystem management [1,2,3,4]. The development of lake eutrophication models has gone from the simple models of single layer, single component, and zero dimension to complex multi-layer, multi-component, three dimensional models [5,6,7,8]. According to the complexity characteristics, lake eutrophication models are divided into simple regression models, simple nutrient balance models, complex water quality, ecological, hydrodynamics comprehensive models and ecological structural dynamics models [9,10,11]. With the advances in computing, monitoring, and communication technologies, simulation and forecasting technologies for water environment of river and lake are also constantly improving [12,13].

Eutrophication is one of the global water environment problems. The main symbol of eutrophication is the abnormal proliferation of algae in water, while the dynamic changes of algae in water are affected by their internal physiological characteristics and external drivers. The growth of algae is affected not only by external factors such as sunlight, nutrients, transparency, water temperature and pH value, but also by hydrodynamic conditions in the water body, such as velocity [14,15], flow [16] and water disturbances [17,18]. The eutrophication of lakes is closely related to human activities in the basin. Humans damage the natural ecological environment through lake reclamation, lakeshore construction and aquaculture, thus increasing the import of nutrients [19,20,21,22]. Regional overall control and classification control should be implemented to control eutrophication in lakes [23].

In the face of agricultural non-point source pollution, applying agricultural non-point source pollution management, nutrient transfer management and soil erosion control is very significant [24]. Some scholars used sediment dredging and source interception methods to improve water quality [25,26]. Eutrophication control of water bodies is beneficial not only to Nature, but also to the survival and development of human beings [27,28,29]. A common phenomenon associated with eutrophication of lakes is the abundance of phytoplankton, especially those that have buoyancy or exercise capacity [30]. In the formation of algae bloom, the Chl-a concentration is generally above 10 mg/m^3^. Due to the widespread presence of algae blooms in freshwater ecosystems and the resulting series of serious water environmental problems, microcystis blooms have received the highest attention as the most studied algal blooms [31]. There are numerous reports on this phenomenon from all over the world [32]. At present, most views agree that the formation of cyanobacteria blooms is generally caused by the physiological characteristics of cyanobacteria and the environmental factors such as temperature, light, nutrients, and other organisms [33]. With the eutrophication of lakes, especially the increase of phosphorus concentration, the species composition of phytoplankton usually leads to the formation of algae bloom. The ratio of total nitrogen to total phosphorus (TN:TP) in water also significantly affects the phytoplankton species composition. Generally, cyanobacterial blooms are dominant when TN:TP < 29 [34]. In the early stages of lake eutrophication, phosphorus is the limiting factor for the algae growth, and its increased concentration leads to the massive growth of algae. With the rapid growth of urban population and the rapid development of industrial and agricultural production in China, the eutrophication of urban water bodies has become increasingly serious and has become a serious ecological problem of urban environment [35,36,37]. The concentration of dissolved oxygen in water decreases, which can cause the death of aquatic animal, especially fish [38,39,40,41]. The final development of eutrophication will make the storage capacity of water body decrease. Due to the deposition of organic residue, the ecological structure of water body will be destroyed, the biological chain will break, the species will tend to be single and the water body function will degenerate. Green carpets formed by eutrophic water make water muddy and reduce transparency. Some algae emit stench and poisonous gases are generated during the anaerobic process of water. These processes greatly affect the sensory impressions of people.

The main research issue in this paper is to plan an artificial playground on original industrial land, and evaluate the water environment of the playground, and suggest control measures. Firstly, the original industrial land was investigated for pollution sources and water quality evaluation, and anti-seepage measures were carried out on the excavation lake area. The TADI method is used to predict the pollution source of the planned artificial playground, and the original river planning scheme is calculated by using the mathematical model of the water environment. The calculation results showed that the original river planning scheme was not suitable. This paper proposed a new “unilateral” river and a new “bilateral” river scheme. It is proposed to improve the water quality of the artificial playground by means of water diversion. Considering the different water levels of the Yangtze River in different seasons, sluice diversion is used to divert water when the water level is high, and pump diversion is used to divert water when the water level is low. Considering that eutrophication in a slow water flow area at a high temperature is easy, a push pump method is proposed to accelerate the flow of water to improve water quality. The flow fields and water quality of unilateral river and bilateral river were analyzed and compared under different circumstances. Considering the project cost, tourism efficiency and environmental benefits, the bilateral river channel is finally determined as the optimal solution. Finally, a comprehensive scheduling scheme was developed for the operation of artificial playgrounds under different conditions.

## 2. Study Area and Methods

### 2.1. Study Area

#### 2.1.1. Study Area Overview

Located at 119°28′ E and 32°15′ N, Zhenjiang City has a monsoon climate with a transition from the warm temperate zone to the northern subtropical zone, belonging to the semi-humid zone. The average annual precipitation is 1082.7 mm. The average annual evaporation is 894.6 mm. The maximum daily evaporation during the year generally occurs in July and August [42]. The minimum evaporation usually occurs in January of each year. Over the years, the average temperature is 15.4 °C, the highest temperature is 40.9 °C, and the lowest temperature is −12 °C. The sunshine is sufficient, with the average annual sunshine hours of 2073.8 h [43]. The annual sunshine percentage is 47–49%. According to the statistics, the annual prevailing winds are E, ENE and ESE (9% each), the annual summer prevailing wind direction is ESE (13%), and the multi-year winter prevailing wind direction is ENE (9%). The prevalent wind of Zhenjiang City is from northeast to east by south. The average wind speed is high, with the annual average wind speed of 3.4 m/s [44].

**Figure 1:**
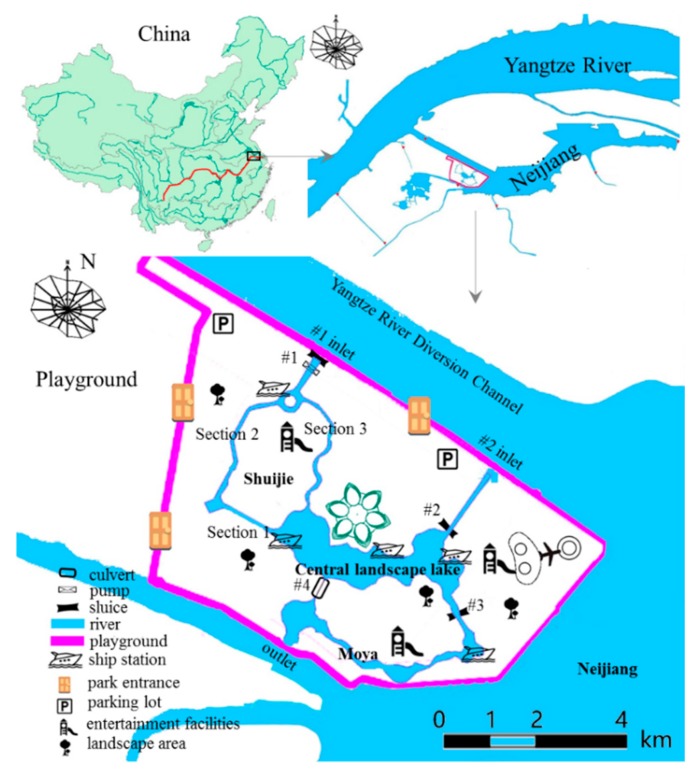
Study area.

Zhenjiang Magic Ocean World project is surrounded by water on three sides, north of the navigation channel west coast, south of the original pilot diversion river, southeast of Neijiang Lake. The water system of Playground Lake, the water conservancy project and surrounding land use situation are shown in Figure 1. The total construction area of Zhenjiang Magic Ocean World project is 101,050 m^2^ [45]. The main construction items include Playground Lake, Ocean World, Water Tourism Center and other tourism facilities, among which the Playground Lake is made up of #1, #2 and #3 sections of Shuijie, central landscape lake and Moya, flood control runs and ecological dispatch through three sluices and one culvert [46].The average COD concentration of Playground Lake is 3.03 mg/L, the average ammonia nitrogen concentration is 0.15 mg/L, the mean total phosphorus concentration is 0.039 mg/L, the mean total nitrogen concentration is 1.182 mg/L and the average Chl-a concentration is 0.016 mg/L. According to Surface Water Class III standards [47,48], the concentration of COD, ammonia nitrogen and Chl-a meet the standards; the standard rates of total phosphorus and total nitrogen are exceeded by 27.3% and 66.7%, respectively.

#### 2.1.2. Study Area Pollution Survey and Water Quality Assessment

**Figure 2:**
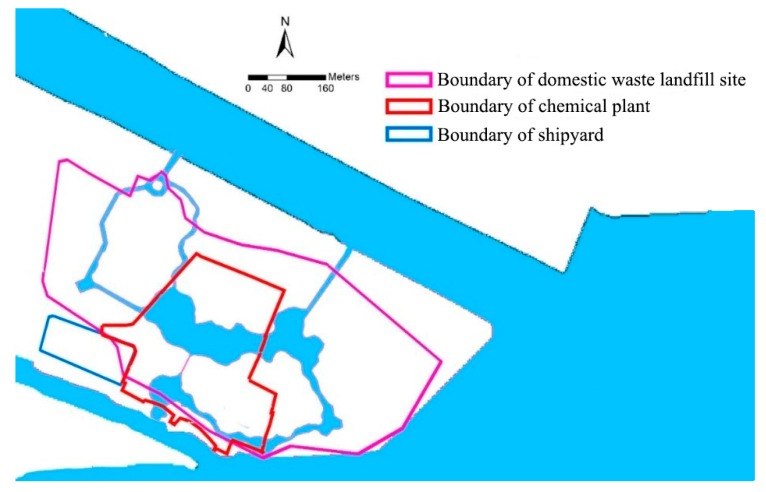
Chemical plant, domestic waste landfill site and shipyard area.

At present, there are mainly three pollution areas in the study area, namely Chemical Plant, Shipyard, and Domestic Waste Landfill Site. As shown in Figure 2.

The chemical plant, demolished from 2008 to 2009, covers an area of about 170,000 m^2^. From 2011 to 2015, the construction of an asphalt pavement was carried out, and soil exchange and agricultural planting were carried out in some areas. The key pollution areas that may exist in the plant area are the original glyphosate production workshop, the phosphonic acid production workshop, the 10% glyphosate aqueous solution production workshop and the sewage treatment station. Landfill covers an area of about 200,000 m^2^, mainly used for domestic waste and a small amount of construction waste, with emphasis on groundwater pollution monitoring. This place contains toxic and hazardous substances, mainly volatile organic compounds, semi-volatile organic compounds, heavy metals, and total petroleum hydrocarbons. The shipyard covers an area of about 27,000 m^2^, and the plant was mainly for machining, and the production of small vessels. The key toxic and hazardous substances of the shipyard are mainly volatile organic compounds, semi-volatile organic compounds, heavy metals and total petroleum hydrocarbons. Based on the results of the preliminary sampling, a total of 139 soil test sites were found in the chemical plant area, and pollutants were detected at all sites. A total of 95 soil contaminants were detected in soil testing, with a detection rate of 72%. There are 38 pollutants that exceeded the screening value. A total of 62 kinds of groundwater pollutants were detected, and 25 kinds of pollutants exceeded the screening value. After the implementation of the playground project, all chemical plants will be cleaned up. Therefore, this study does not consider the impact of the chemical plant on the project. According to the preliminary investigation and analysis of the site environment, the detection indexes of domestic waste landfill soil are generally good. Most of the indicators did not exceed the selection of environmental risk evaluation screening standards (the garbage layer was not analysed), groundwater environmental quality standards greatly exceed the standard of Class III [47,48], subject to a greater degree of pollution. After demolition was completed in 2009, the shipyard was covered by imported earth filling and construction waste and has remained idle till now. The wharf is far away from the wading area of the project and is free from water pollution according to this study. According to the plan, the total excavation earthwork within the scope of soil treatment is 300,000 m^3^, the earthwork to be treated is 220,000 m^3^, and the remaining 80,000 m^3^ shall be earthwork without treatment.

### 2.2. Study Methods

#### 2.2.1. Hydrodynamic Two-Dimensional Model

The Cartesian coordinate system of two-dimensional hydrodynamic governing equations is the continuity equations and momentum equations for the integral of the three-dimensional Renault Navier-Stokes equations [2,3] of the incompressible fluid along the direction of water depth, which can be expressed as follows:

(1) $$\mathrm{Continuity}\;\mathrm{equation}:\;\frac{\partial h}{\partial t}+\frac{\partial h\bar{u}}{\partial x}+\frac{\partial h\bar{v}}{\partial y}=hQ$$

Momentum equation:

(2) $$\frac{\partial h\bar{u}}{\partial t}+\frac{\partial h{\bar{u}}^{2}}{\partial x}+\frac{\partial h\bar{vu}}{\partial y}=fh\bar{v}-gh\frac{\partial \eta }{\partial x}-\frac{h}{\rho_{0}}\frac{\partial P_{a}}{\partial x}-\frac{gh^{2}}{2\rho_{0}}\frac{\partial \rho }{\partial x}+\frac{\tau_{sx}}{\rho_{0}}-\frac{\tau_{bx}}{\rho_{0}}-\frac{1}{\rho_{0}}\left(\frac{\partial S_{xx}}{\partial x}+\frac{\partial S_{xy}}{\partial y}\right)+\frac{\partial }{\partial x}\left(hT_{xx}\right)+\frac{\partial }{\partial y}\left(hT_{xy}\right)+hu_{s}Q$$

(3) $$\frac{\partial h\bar{v}}{\partial t}+\frac{\partial h{\bar{v}}^{2}}{\partial y}+\frac{\partial h\bar{vu}}{\partial x}=-fh\bar{u}-gh\frac{\partial \eta }{\partial y}-\frac{h}{\rho_{0}}\frac{\partial P_{a}}{\partial y}-\frac{gh^{2}}{2\rho_{0}}\frac{\partial \rho }{\partial y}+\frac{\tau_{sy}}{\rho_{0}}-\frac{\tau_{by}}{\rho_{0}}-\frac{1}{\rho_{0}}\left(\frac{\partial S_{yx}}{\partial x}+\frac{\partial S_{yy}}{\partial y}\right)+\frac{\partial }{\partial x}\left(hT_{xy}\right)+\frac{\partial }{\partial y}\left(hT_{yy}\right)+hv_{s}Q$$

where: *t* represents time; *x*, *y* represent Cartesian coordinates; *h* represents total water depth; *η* represents water level; *ρ* represents water density; $\bar{u}$ and $\bar{v}$ represent average water depth; *f* = 2*Ωsinφ* denotes the Coriolis factor (*Ω* is the angular velocity of the Earth’s rotation, *φ* is the geographical latitude); $S_{xx}$, $S_{xy}$ and $S_{yy}$ are the radiation stress tensors; *P_a_* is the atmospheric pressure; *Q* is the point source emissions; g is the gravitational acceleration:

(4) $$h\bar{u}=\int_{-d}^{\eta }udz,h\bar{v}=\int_{-d}^{\eta }vdz$$

where: $\rho_{0}$ represents the relative density of water; $\left(u_{s},v_{s}\right)$ represents the rate at which the outside world is released into the waterbody.

Transverse stress *T_ij_* includes viscous resistance, turbulent frictional resistance, and differential advection frictional resistance, which can be calculated using the eddy viscosity equation of the mean vertical velocity:

(5) $$T_{xx}=2A\frac{\partial \bar{u}}{\partial x},\;\;T_{xy}=A\left(\frac{\partial \bar{u}}{\partial y}+\frac{\partial \bar{v}}{\partial x}\right),\;\;T_{yy}=2A\frac{\partial \bar{v}}{\partial x}$$

#### 2.2.2. Water Quality and Eutrophication Two-Dimensional Model

(1) Basic equations of water quality model

The water quality equation is based on the mass balance equation. The three-dimensional water quality transport equation contains a lot of uncertain parameters. Under the existing conditions, the verification of the model is difficult. Considering the factors such as data and model calculation workload, the average vertical two-dimensional water quality model is adopted [49,50]. Two-dimensional water quality transport equation is:

(6) $$\frac{\partial C_{i}}{\partial t}+U\frac{\partial C_{i}}{\partial x}+V\frac{\partial C_{i}}{\partial y}=\frac{\partial }{\partial x}\left(E_{x}\frac{\partial C_{i}}{\partial x}\right)+\frac{\partial }{\partial y}\left(E_{y}\frac{\partial C_{i}}{\partial y}\right)+K_{i}C_{i}+S_{i}$$

where: *C_i_* is the pollutant concentration; *u*, *v* are the flow velocity components in the *x* and *y* directions, respectively; *E_x_* and *E_y_* are the diffusion coefficients in the *x* and *y* directions, respectively; *K_i_* is the pollutant degradation coefficient; *S_i_* is the pollutant sediment release item. 

In order to introduce a quantitative relationship between sediment resuspension flux and hydrodynamic conditions in the model and reflect the change of resuspension flux of each pollutant in the sediment with the flow velocity. The sediment resuspension flux is calculated using the relationship obtained from sediment resuspension experiments when establishing the mathematical model [49,50], which mainly reflects the handling of the source sink term Si, as follows:

(7) $$S_{i}=\frac{\alpha_{i}}{H}$$

where: $\alpha_{i}$ is the sediment resuspension flux (g/(m^2^∙d)), $\alpha_{i}=\zeta_{i}\cdot \beta_{i}\exp\left(\xi_{i}\cdot P\right)$; *H* represents water depth (m); *β_i_* is the proportion of sediment pollutants in SS (%); P represents co-velocity (cm/s), $P=\sqrt{u^{2}+v^{2}}$; $\zeta_{i}$, $\xi_{i}$ are the sediment resuspension parameters.

(2) Basic equations of Ecolab eutrophication model

The content of Chl-a in lakes is the major parameter for evaluating the water trophic status. There are many factors affecting Chl-a content in lakes. It is generally acknowledged that light, temperature, precipitation, nutrients and pH affect it. In this paper, the impact of nutritive salts (total nitrogen, total phosphorus) concentrations on algae growth were investigated. Chl-a concentration was regarded as the evaluation index. According to the principle of mass conservation, the basic equation of eutrophication variables is [36,37,38,39,40,41]:

(8) $$\frac{\partial C_{c\mathrm{hl}-a}}{\partial t}+U\frac{\partial C_{\mathrm{chl}-a}}{\partial x}+V\frac{\partial C_{\mathrm{chl}-a}}{\partial y}=\frac{\partial }{\partial x}\left(E_{x}\frac{\partial C_{\mathrm{chl}-a}}{\partial x}\right)+\frac{\partial }{\partial y}\left(E_{y}\frac{\partial C_{\mathrm{chl}-a}}{\partial y}\right)+S_{\mathrm{chl}-a}$$

where:

(9) $$S_{chl-a}=G_{PI}\left(t\right)-D_{PI}\left(t\right)-\frac{V_{s}}{D}$$

(10) $$G_{PI}=K_{1}\cdot Phtsy\cdot F\left(N,P\right)$$

(11) $$D_{PI}=\mu \cdot F\left(N,P\right)$$

In the equation, *C*_chl-a_ represents the concentration of Chl-a; *U* and *V* respectively represent the flow velocity components in the *x* and *y* axis directions, which can be calculated from the water volume model; *E_x_* and *E_y_* represent the lateral and longitudinal diffusion coefficients of algae, respectively; *S*_chl-a_ represents the conversion of Chl-a; *G_PI_* represents the algal growth; *D_PI_* represents the algal death; *V_s_* represents the algal sedimentation; *D* represents the water depth; K_1_ refers to the correlation coefficient between Chl-a content and photosynthesis of phytoplankton; Phtsyn refers to the photosynthesis value of plants in unit water volume; *μ* refers to the mortality rate under optimal nutrition conditions; *F* (*N*, *P*) indicates the nutrient limit function:

(12) $$F\left(N,P\right)=\frac{2}{\frac{1}{F\left(N\right)}+\frac{1}{F\left(P\right)}}$$

(13) $$F\left(N\right)=\frac{\frac{PN}{PC}-PN_{min}}{PN_{max}-PP_{min}}$$

(14) $$F\left(P\right)=\frac{\left(\frac{PP}{PC}-PP_{min}\right)\cdot \left(KC+PP_{max}-PP_{min}\right)}{\left(PP_{max}-PP\right)\cdot \left(KC+PP/PC-PP_{min}\right)}$$

where $PN_{min}$ and $PN_{max}$ are respectively the minimum and maximum nitrogen content of algae (gN/gC). $PP_{min}$ and $PP_{max}$ are the minimum and maximum values of phosphorus content in algae, respectively (gP/gC). KC is the phytoplankton half-saturation content of phosphorus (gP/gC).

#### 2.2.3. Pollution Source Prediction after the Completion of the Playground

After the chemical plant clean-up completion, landfills are also all clean. The ground is also cleared of pollutants in the Shuijie commercial area, Sea World and Polar Dry Skiing areas. Only travelers will be exposed to some source of pollution in all aspects of tourism. The exposed population in this project mainly comes from two sources: one is the tourist population from other places; the other is the residents nearby. Based on the analysis of market penetration of the same type of tourism products, it is concluded that the capture rate of the project to the residential market is about 15% and that of the tourist market is about 6%. The number of tourists in this project is about 1.7 million/a. According to the prediction formula of tourist quantity, the traffic flow of local residents is about 1.03 million/a. The number of foreign visitors is about 670,000/a and the peak daily passenger flow is 6000/a.

(1) Index construction

Emission of pollutants from tourism activities (S): affected by such factors as the per capita water consumption of tourism, sewage discharge coefficient, sewage treatment rate, pollutant concentration and other factors. Emissions of tourism activities can be calculated using the following formula:

(15) $$S=W\cdot r\cdot u\cdot C_{D}+W\cdot r\left(1-u\right)C_{w}$$

where S is the discharge of pollutants (such as tourism accommodation activities, tourism catering activities, excursions, etc.); W is the per capita water consumption; r is the sewage discharge coefficient; and u is the sewage treatment rate; $C_{D}$ represents the concentration of pollutants after sewage treatment; $C_{w}$ represents the concentration of pollutants before sewage treatment.

Tourist Activities Disturbance Index, TADI: The indexes of total nitrogen (TN), total phosphorus (TP), five days biochemical oxygen demand (BOD_5_) and chemical oxygen demand (COD) were selected to construct a comprehensive evaluation index of pollutant emissions of tourists. Due to the different evaluation criteria of various types of pollutant emissions, in order to synthesize various types of pollutant emissions evaluation results, each type of pollutant emissions must be non-dimensional treatment. First, the indicators are standardized mean difference, and then use the weighted arithmetic average method to construct tourist comprehensive evaluation index of pollutant emissions. Among them, the range of standardization formula is as follows:

(16) $$M_{i}=\frac{S_{i}-S_{min}}{S_{max}-S_{min}}$$

where $M_{i}$ is the standard value of pollutants; $S_{i}$ is the pollutant index value; $S_{min}$ is the minimum pollutant index value; $S_{max}$ is the maximum pollutant index value.

The TADI is calculated as follows:

(17) $$\mathrm{TADI}=\overset{m}{\underset{j=1}{\sum }}\overset{n}{\underset{i=1}{\sum }}M_{i}/m$$

where M is the standard value of pollutants; i is the type of tourism activity subscript, i=1, 2, 3… n, that there are n kinds of tourism activities; j is the pollutant type subscript  j=1, 2, 3, 4. TADI, is in the range of [0, 1]. Based on the range of pollutant emission intensity and application, TADI can be divided into five basic evaluation grades of 1, 0.8, 0.6, 0.4, 0.2. Grade 1 value below 0.2, low-interference type; grade 2 value between 0.2 and 0.4, lower interference type; grade 3 value between 0.4 to 0.6, moderate interference type; grade 4 value between 0.6 and 0.8, higher interference type; grade 5 value above 0.8, highly interference type. The calculated concentration of pollutants in the main part of the playground is shown in Table 1.

### 2.3. Model Setup and Parameters Selection

#### 2.3.1. Model Setup

**Figure 3:**
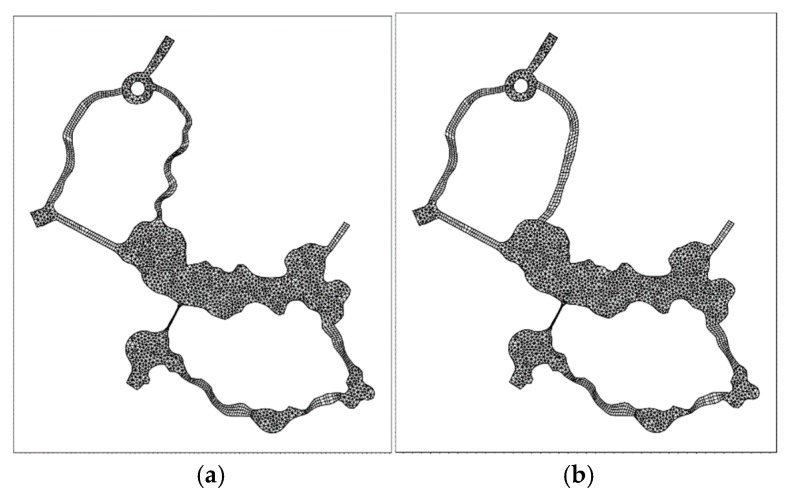
(**a**) Original planning “bilateral” river grid, (**b**) proposed “bilateral” river grid.

**Figure 4:**
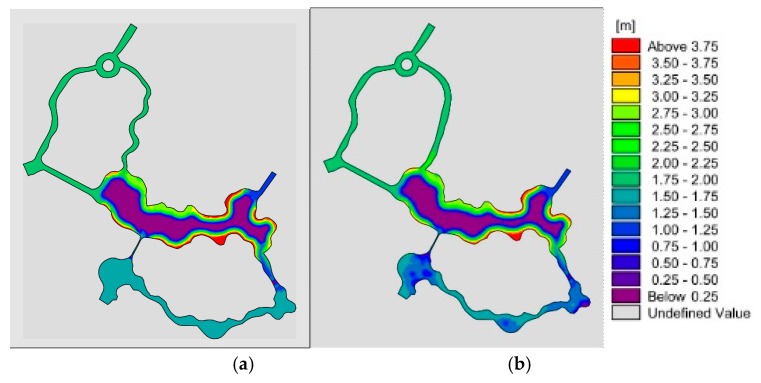
(**a**) The original planning of “bilateral” river elevation, (**b**) recommended “bilateral” river elevation.

In the model calculation, the Magical Marine World in Zhenjiang is divided into a three-quadrangle mixed grid with a grid spacing of about 8–10 m. Suppose the initial time the lake is stationary, there is no disturbance, the time step Δt = 60 s. The original planning “bilateral” river, the proposed “bilateral” river grid model and topographic elevation shown in Figure 3 and Figure 4. 

The wading area elevation as a whole changed within 0–3 m, of which the deepest lake center, the central elevation of 0 m, Moya area at about 1.5 m, Shuijie area at about 2 m. According to the actual topography of the bwading area, the geology and geographical location of the model, the Manning coefficient is 38 m^1/3^/s and the eddy parameter is 0.28. See the Appendix A for initialization parameters.

#### 2.3.2. Parameters Selection

Due to the current planning state and no excavation operations on the playground, the model calculation results were checked according to Wang [35,36,37,38], in situ data and routine monitoring data of Neijiang Lake and diversion channel, in order to meet more the strong relation between the real status of the park and the model to estimate the effect of changes. The model water quality and eutrophication parameters are listed in Table 2.

### 2.4. Water Diversion Program, River Layout and Seepage Prevention Area

#### 2.4.1. Water Diversion Program

(1) Water diversion through sluice

**Figure 5:**
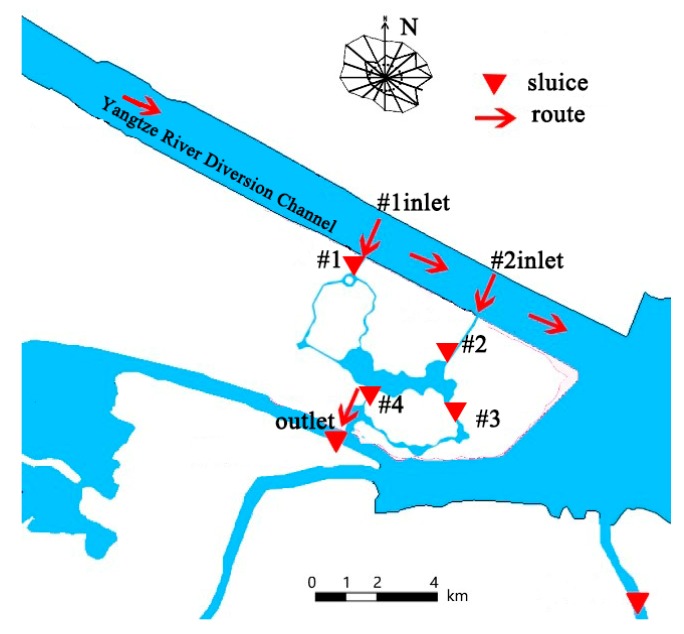
Route of water diversion through the sluice.

During the high tide of spring-tide of the Yangtze River, the water level of the Yangtze River is higher than that of the Neijiang Lake, with a 0.3 m sluice. Water will be diverted into the Playground Lake by gravity to improve water quality and eutrophication by the use of water head between the #1 inlet, #2 inlet and outlet (Figure 5).

Model boundary conditions: The initial water level was set at 2.67 m. The temperature was 28 °C. The initial flow rate was set to zero [42,43,44]. The initial water head Δh (the water head between the Yangtze River and the Playground Lake) was 0.2–0.3 m. The flow rate of water diversion through sluice at #1 inlet and #2 inlet were 8.27 and 1.82 m^3^/s, respectively.

Weather conditions: The actual measurement data show that the average temperature in 2016 is 16.8 °C; the coldest month is January, the average monthly temperature is 2.97 °C; the hottest month is August, the average monthly temperature is 29.39 °C. The average annual precipitation in recent two years is 1082.7 mm, which is unevenly distributed. The precipitation is mostly concentrated in the spring, summer and autumn seasons [45,46]. Especially, the precipitation is the highest in the summer, exceeding 45% of the total annual precipitation. The average annual wind speed is 3.4 m/s.

Initial nutrient salts and pollution sources load: The amount of emissions by tourists and sewage produced by tourism facilities were calculated as pollution sources load, according to the measured data and index construction method [48].

(2) Water diversion through Pump

**Figure 6:**
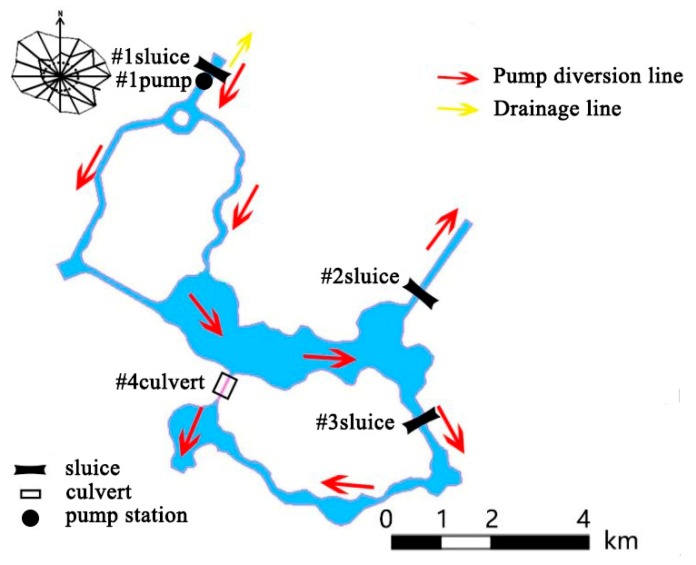
Route of water diversion through pump.

Through the #1 pumping station, the diversion water will enter the playground to improve its water quality and eutrophication status. The position of “three sluices and one culvert” is shown in Figure 6. The specification parameters are given in Table 3.

The Playground Lake has a storage capacity of about 350,000 m^3^. The design flow of a single pump for the #1 pump is 1.85 m^3^/s. water can be changed once within 27 h by a double pump diversion, according to the initial calculation. The pump diversion will not affect recreational activities such as watercraft due to its low flow, so that continuous pump diversion can be used. In order to ensure that the water is completely replaced, the proposed pumping duration is 30 h. If we turn off the #1 sluice, turn on the #1 pump, #2 sluice, #3 sluice and #4 culvert to divert water, most of the water will flow out from #2 sluice, and the water in the Moya area cannot be effectively changed. Therefore, it is necessary to properly allocate “three sluices and one culvert” to make the water completely replaced. As the ratio of the storage capacity of the Shuijie area and central landscape lake region to the Moya area is about 3:1, the pumping method design is from 0:00 to 22:00 on the first day (lasting 22 h) to change water in the Shuijie area and central landscape lake region, and from 22:00 on the first day to 6:00 on the second day (lasting 8 h) to change water in the Moya area (Table 4). 

(3) Water cycle through push pump

**Figure 7:**
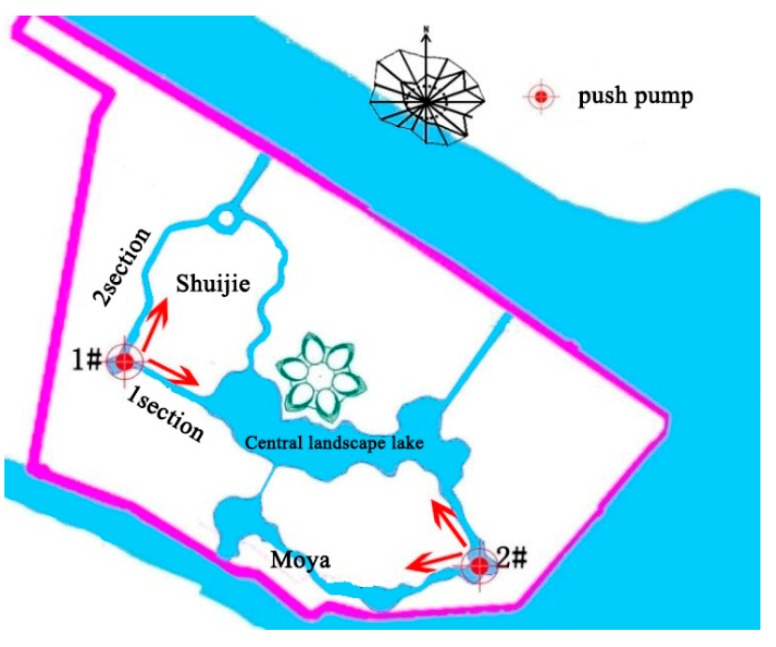
Push pump position.

Due to the stagnation phenomenon of the partial wading area, we consider the use of a push pump at the corner of the Shuijie 1–2 section and Moya area (Figure 7). The design flow of the push pump is 0.2 m^3^/s. The stream is pushed through to improve the Shuijie 1–2 section and Moya area of the water cycle. According to the algae outbreak data and preliminary calculations, algae basically do not appear when the temperature is less than 15 °C, and we do not open the push pump when the temperature is less than 15 °C. When the temperature is greater than 15 °C, #1 pump and sluice are not open, and open the rest of the time. The push pump on and off settings are shown in Table 5.

The model calculation schemes are shown in Table 6. According to the measured meteorological data, the southeaster wind with the highest wind frequency was selected in summer and the average temperature was 28 °C. The flow field, water quality and eutrophication of the wading area under no water diversion, pump diversion and sluice diversion were simulated, respectively. The northeast wind with the highest wind frequency was selected in winter and the average temperature was 7 °C. The flow field, water quality and eutrophication of the wading area under no water diversion and pump diversion were simulated respectively.

#### 2.4.2. River Layout

(1) The original planning layout problems

**Figure 8:**
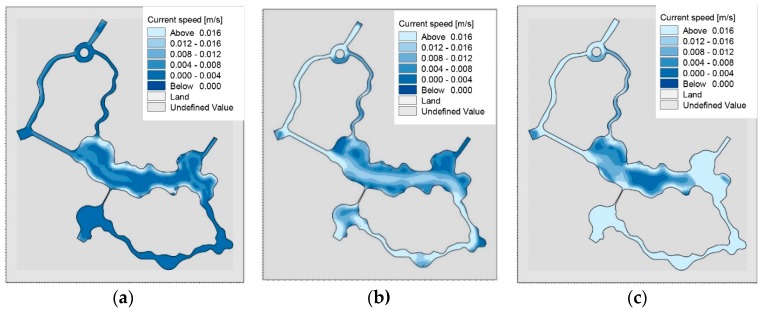
(**a**) Flow field without water diversion; (**b**) Flow field after pump diversion 30 h; (**c**) Flow field after sluice diversion.

Based on the constructed playground lake water mathematical model, the flow field in the lake area under no water diversion, pump diversion 30 h and sluice diversion conditions were calculated respectively (Figure 8a–c). 

It can be seen from the figures that the overall flow rate of the entire wading area is small under the condition of no water diversion, with an average velocity of 0.003 m/s. Only the center of the lake is shallow at the edge of the lake and the flow velocity is relatively fast. Pump and sluice diversion of the overall flow field have significantly improved. However, due to the meandering distribution of the waterway in the Shuijie 3 zone and the narrow width of the estuary (12~20 m), the flow field in the Shuijie 3 area is not smooth. Further, a large area of stagnant area will be formed in the Shuijie 3 part connected to the central landscape lake. Coupled with the large flow of people here this caused a greater point source pollution.

(2) The new plan of the river layout program

**Figure 9:**
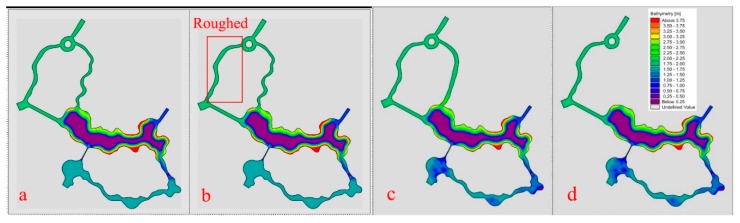
The river layout program. (**a**) Original Planning river; (**b**) “Roughed” river; (**c**) “Bilateral” rivers; (**d**) “Unilateral” river.

In order to improve the flow field in wading area, three kinds of river construction schemes are proposed considering the original river layout and project construction progress:

① Original Planning river: the original planning river layout shown in Figure 9a;
② “Roughed” river: roughly handle the Shuijie 2 and the ring lake (planted with plants, retaining walls and other “soft isolation” devices). To improve the flow ratio of Shuijie 2 and 3, so as to allow more water to enter the Shuijie 3. The layout of the river is shown in Figure 9b.
③ “Bilateral” rivers: smoothing the river channel in the Shuijie 3 to improve the flow ratio of the Shuijie 2 and 3 so as to allow the water to pass through the two rivers evenly. The layout of the river is shown in Figure 9c;
④ “Unilateral” river: remove the wading area north of Shuijie 3, so that all water flows into the Shuijie 2, the river layout shown in Figure 9d.

“Roughed” river project complex, being costly, it is not recommended so the “bilateral” river, and “unilateral” river layout plans are recommended.

#### 2.4.3. River Seepage Prevention Area Determination

**Figure 10:**
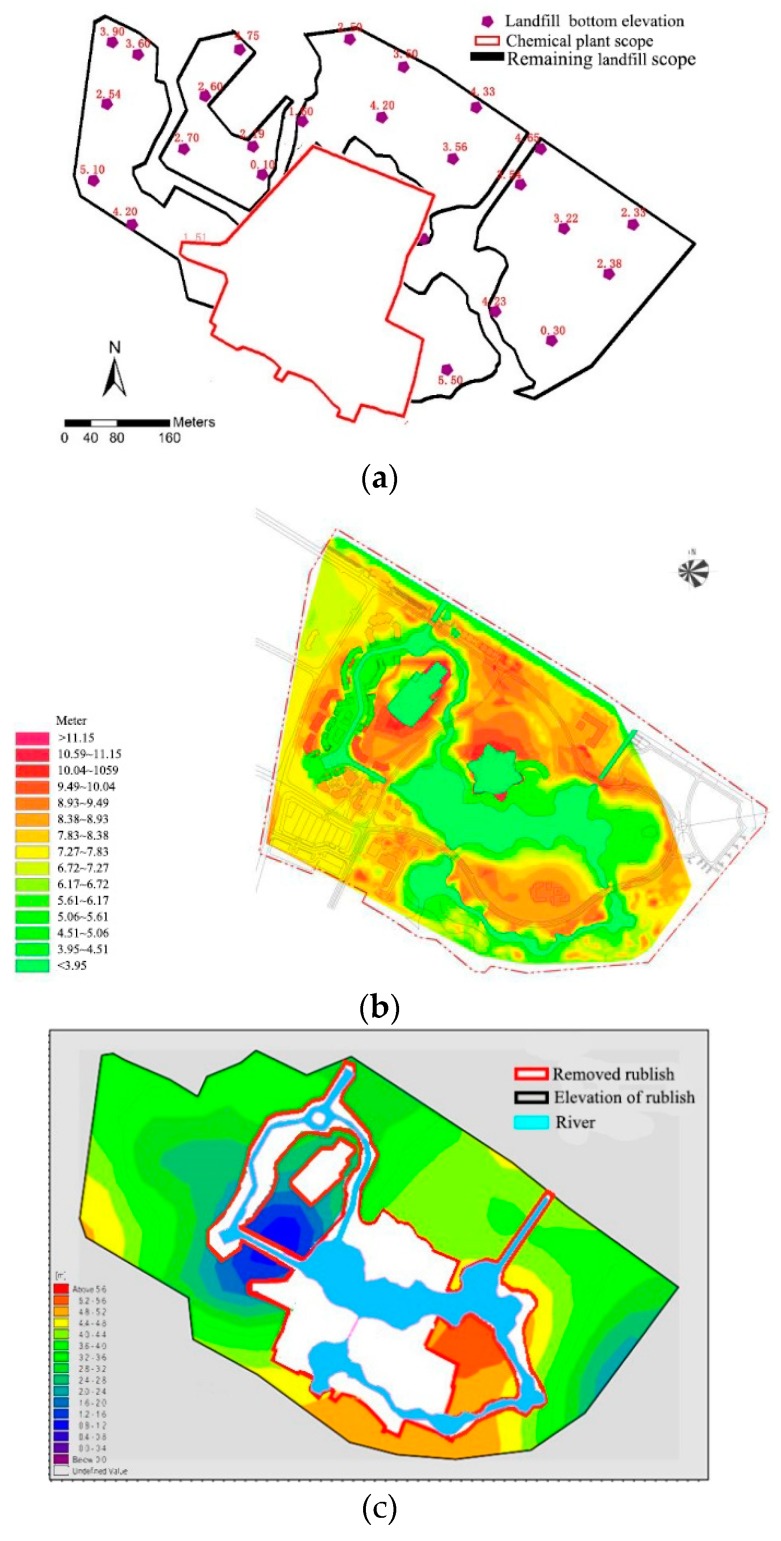
(**a**) The remaining landfill scope and bottom elevation after project implementation; (**b**) Designed ground elevation after infrastructure foundation and river excavation; (**c**) Superimposed recommended “bilateral” rivers remaining landfill range.

After the project is implemented, the landfill where the river is located is excavated. At the chemical plant location the old soil is replaced with new, non-polluting soil backfill. The remaining landfill scope and bottom elevation at the end of the project are shown in Figure 10a. Playground design ground elevations after infrastructure foundation and river excavation are shown in Figure 10b. 

The range and elevation of the remaining landfill overlaying the recommended “bilateral” rivers are obtained by linear interpolation, as shown in Figure 10c. It can be seen from the figure that there is no rubbish in the area where the bottom of the river is located, and only some of the river banks are close to the remaining rubbish. Therefore, there is a risk of pollutants infiltrating into the earth, which may cause water pollution in the wading area.

**Figure 11:**
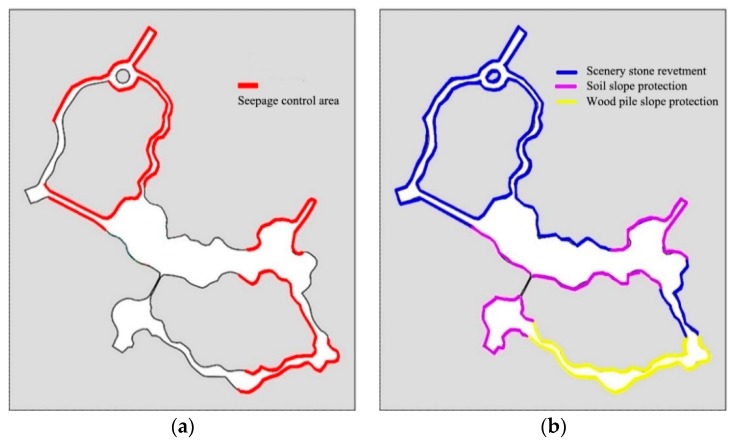
(**a**) River seepage control area. (**b**) River slope protection type.

After the implementation of the project, the chemical plant will be completely cleaned up and the landfill site under the river will be completely cleaned up. The Shuijie business district, Sea World and Polar Dry Skiing ground contaminants have also been cleared. In order to prevent landfill leachate approaching the slope from entering the river water body, seepage prevention is required when the distance between the remaining garbage and the riverway is small (less than 20 m), and the seepage prevention zone is finally determined, as shown in Figure 11a. River slope protection type is shown in Figure 11b.

## 3. Results and Discussion

According to the definition of eutrophication, it is well known that abundant nitrogen, phosphorus and other nutrients, slow flow velocity and suitable climatic temperature are the three essential conditions for the eutrophication. Although the climatic conditions cannot be controlled by man and cannot be described quantitatively, the nutrient content and hydrodynamic conditions in the water body can be controlled. The following two factors are used as variables to predict the eutrophication of water body. Nitrogen and phosphorus nutrients are the most important material basis for algae growth. Chlorophyll (Chl-a) can reflect the status of algae breeding. Therefore, this study proposed the combination of three single indicators of total nitrogen (TN), total phosphorus (TP) and chlorophyll (Chl-a) as the evaluation factors of urban water body landscape water quality.

### 3.1. Flow Field Calculation and Analysis

#### 3.1.1. Analysis of Flow Field without Water Diversion

**Figure 12:**
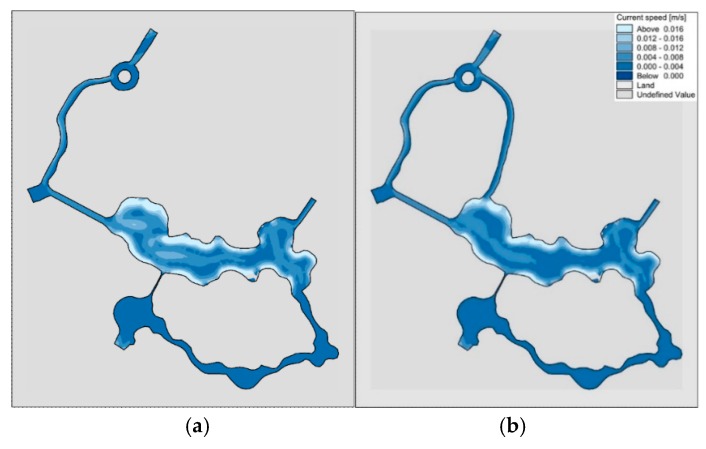
Comparison of flow field in “unilateral” and “bilateral” rivers without water diversion. (**a**) “unilateral” river; (**b**) “bilateral” rivers.

Using the constructed two-dimensional model of the hydrodynamic, the comparison of flow fields in “unilateral” and “bilateral” rivers under no water diversion conditions is shown in Figure 12. It can be seen in the absence of water diversion, the flow velocity in the two rivers is very slow, with little difference.

#### 3.1.2. Analysis of Flow Field with Water Diversion through Pump

**Figure 13:**
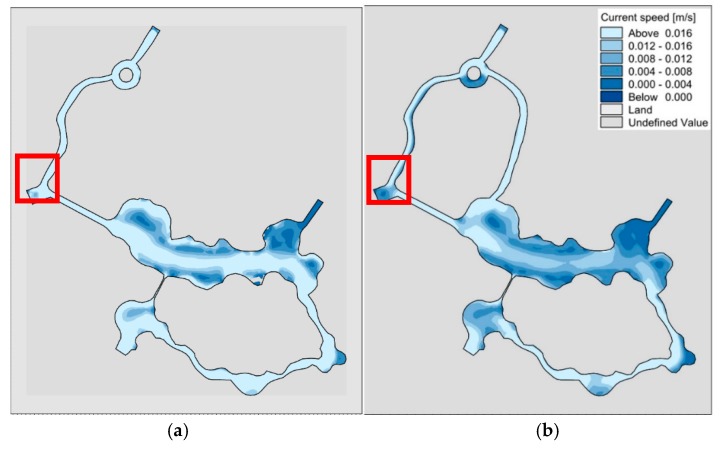
Comparison of the flow field in “unilateral” and “bilateral” rivers after 30 h of pumping. (**a**) “unilateral” river; (**b**) “bilateral” rivers.

Using the constructed two-dimensional hydrodynamic model, the comparison of the flow field between the “unilateral” and “bilateral” channels after pumping for 30 h is shown in Figure 13. 

As can be seen from the figure, under the condition of pumping, due to the reduction of “unilateral” river bank capacity, the pump effect is better than that of “bilateral” river channel, especially in the marked red box mark.

#### 3.1.3. Analysis of Flow Field with Water Diversion through Sluice

**Figure 14:**
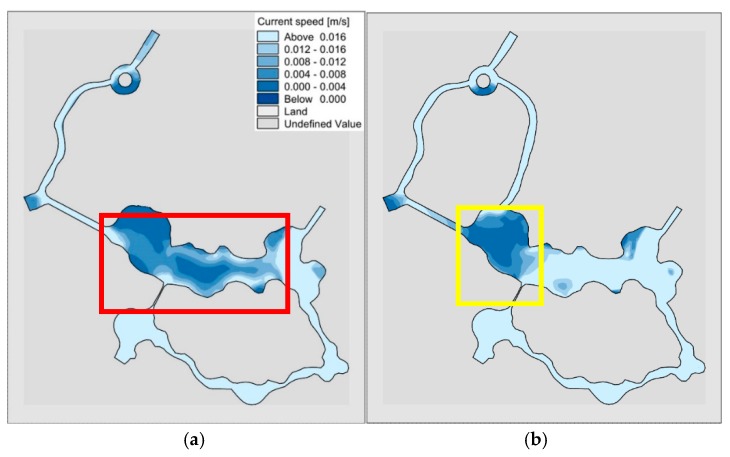
Comparison of flow field in “unilateral” and “bilateral” rivers under sluice diversion conditions. (**a**) “unilateral” river; (**b**) “bilateral” rivers.

Using the constructed two-dimensional hydrodynamic model, we can calculate the flow field of “unilateral” and “bilateral” rivers under the sluice diversion conditions, as shown in Figure 14. As can be seen from the figure, due to the removal of the river in the Shuijie 3 area, the water flow at the location indicated by the red box in the “unilateral” channel is not smooth, and forms a large stagnant area, which is not conducive to the smooth flow of water in the wading area. Due to the sudden widening of the water surface in the lake area from the water block to the central area as shown by the yellow box in the “bilateral” rivers, the stagnation of the reciprocating flow occurs, but it does not affect the overall flow field. This shows that the distribution of flow field in “bilateral” rivers is better than “unilateral” river, so we recommend “bilateral” rivers.

### 3.2. Water Quality and Eutrophication

#### 3.2.1. Analysis of Water Quality and Eutrophication without Water Diversion in Summer

**Figure 15:**
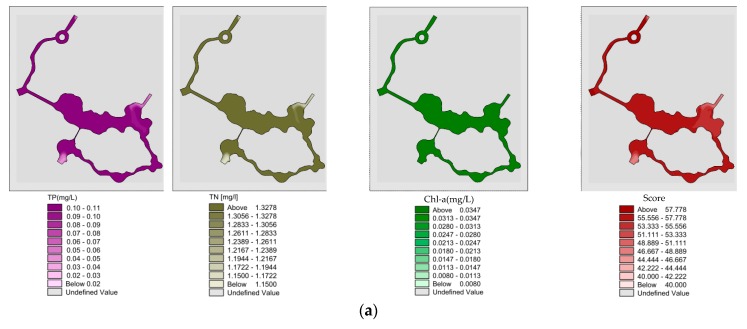

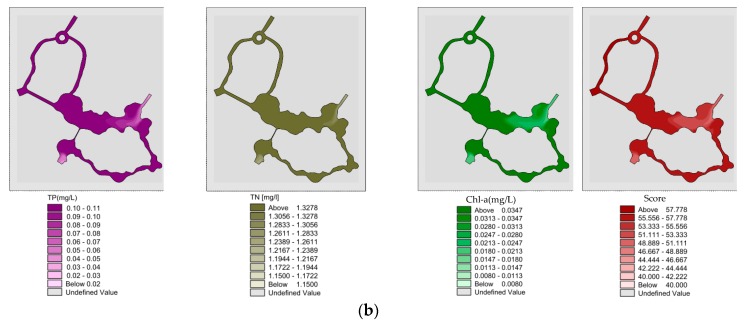
(**a**) Spatial distribution of “unilateral” river TP, TN, Chl-a and eutrophication comprehensive scores without water diversion in summer; (**b**) Spatial distribution of “bilateral” rivers TP, TN, Chl-a and eutrophication comprehensive scores without water diversion in summer.

Spatial distribution of “unilateral” river TP, TN, Chl-a and eutrophication comprehensive scores without water diversion in summer (Figure 15a). The mean value of TP, TN, Chl-a and eutrophication score of the wading area obtained after the “unilateral” river reached a completely steady state after 10 days under a southeastern wind were 0.107, 1.385 and 0.04 mg/L and 57.1, and almost all areas show mild eutrophication; Spatial distribution of “bilateral” rivers TP, TN, Chl-a and eutrophication comprehensive scores without water diversion in summer (Figure 15b). After southeastern wind exposure for 10 days, the TP, TN, Chl-a and eutrophication comprehensive scores obtained after the “bilateral” rivers reached a completely steady state have mean values of 0.106, 1.383 and 0.039 mg/L and 56.7. In addition to the exchange of water inlet and outlet area #2, almost all areas show mild eutrophication.

#### 3.2.2. Analysis of Water Quality and Eutrophication with Pumping Diversion in Summer

**Figure 16:**
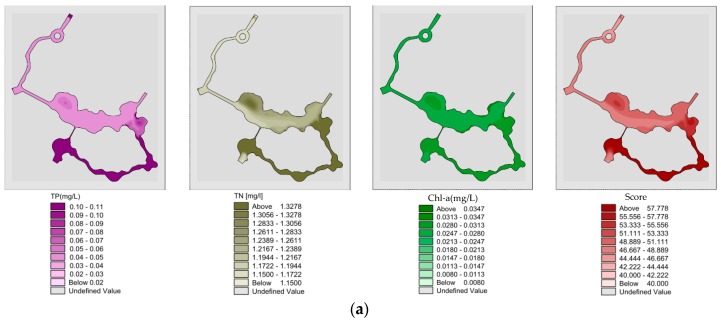

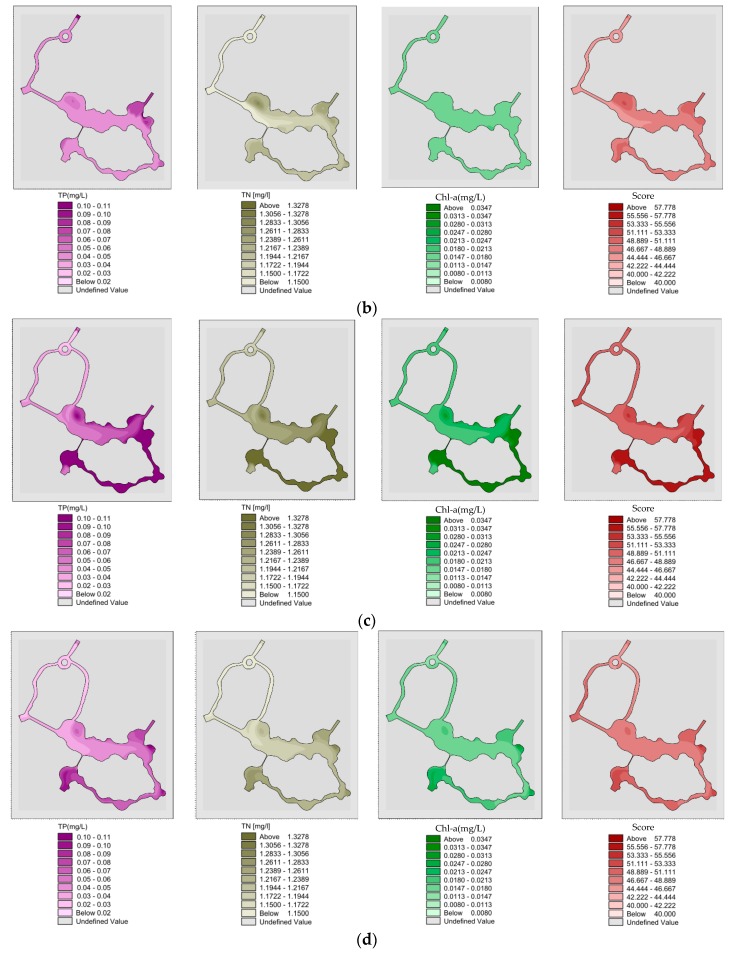
(**a**) Spatial distribution of “unilateral” river TP, TN, Chl-a and eutrophication scores after pumping 22 h in summer; (**b**) Spatial distribution of “unilateral” river TP, TN, Chl-a and eutrophication score after pumping for 30 h in summer, whereby the “unilateral” river model reached a completely steady state; (**c**) Spatial distribution of “bilateral” river TP, TN, Chl-a and eutrophication score after pumping for 22 h in summer; (**d**) Spatial distribution of “bilateral” river TP, TN, Chl-a and eutrophication score after pumping for 30 h in summer.

Spatial distribution of “unilateral” river TP, TN, Chl-a and eutrophication scores after pumping 22 h in summer, after which the “unilateral” river model reached a completely steady state (Figure 16a). The mean values of TP, TN, Chl-a and eutrophication in the wading area were 0.087, 1.296 and 0.028 mg/L and 52.9 respectively; Spatial distribution of “unilateral” river TP, TN, Chl-a and eutrophication score after pumping for 30 h in summer, whereby the “unilateral” river model reached a completely steady state (Figure 16b). The mean values of TP, TN, Chl-a and eutrophication in wading area were 0.05, 1.203 and 0.017 mg/L and 48.5; Spatial distribution of “bilateral” river TP, TN, Chl-a and eutrophication score after pumping for 22 h in summer (Figure 16c). After the 22 h pumping, the “bilateral” river model reaches a completely steady state. The mean values of TP, TN, Chl-a and eutrophication score in wading area were 0.086, 1.294 and 0.027 mg/L and 52.7, respectively; Spatial distribution of “bilateral” river TP, TN, Chl-a and eutrophication score after pumping for 30 h in summer (Figure 16d). The model reached a fully steady state after these 30 h of pumping. The mean values of TP, TN, Chl-a and eutrophication score in the wading area were 0.05, 1.201 and 0.017 mg/L and 48.4 respectively.

#### 3.2.3. Analysis of Water Quality and Eutrophication with Sluice Diversion

**Figure 17:**
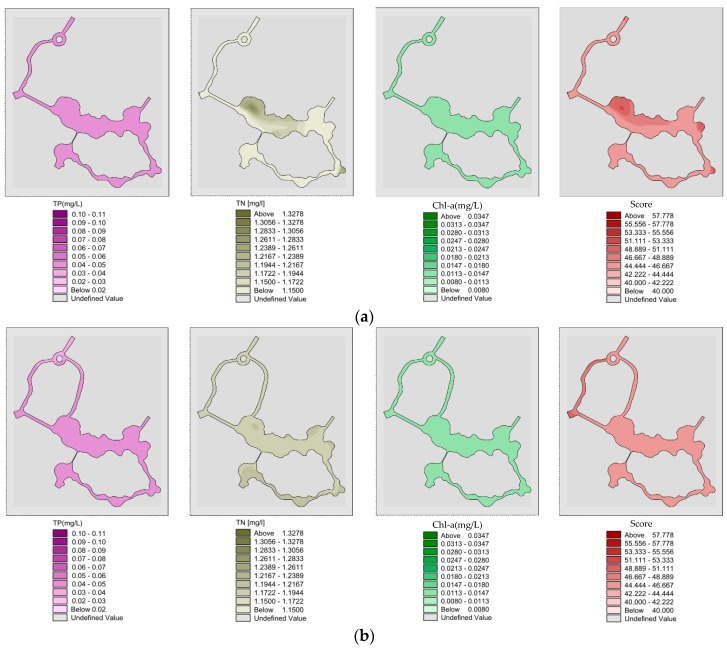
(**a**) Spatial distribution of “unilateral” river TP, TN, Chl-a and eutrophication scores by sluice diversion in summer; (**b**) Spatial distribution of “bilateral” river TP, TN, Chl-a and eutrophication scores by sluice diversion in summer.

Spatial distribution of “unilateral” river TP, TN, Chl-a and eutrophication scores by sluice diversion in summer (Figure 17a). After the “unilateral” river model has reached a completely steady state under the sluice diversion conditions, the mean values of TP, TN, Chl-a and eutrophication score in wading area were 0.04, 1.202 and 0.016 mg/L and 46.3, respectively. Spatial distribution of “bilateral” river TP, TN, Chl-a and eutrophication scores by sluice diversion in summer (Figure 17b). After the “bilateral” rivers model has reached a completely steady state under the sluice diversion conditions, the mean values of TP, TN, Chl-a and eutrophication score in wading area were 0.04, 1.191 and 0.012 mg/L and 45.3, respectively.

#### 3.2.4. Analysis of Water Quality and Eutrophication without Water Diversion in Winter

**Figure 18:**
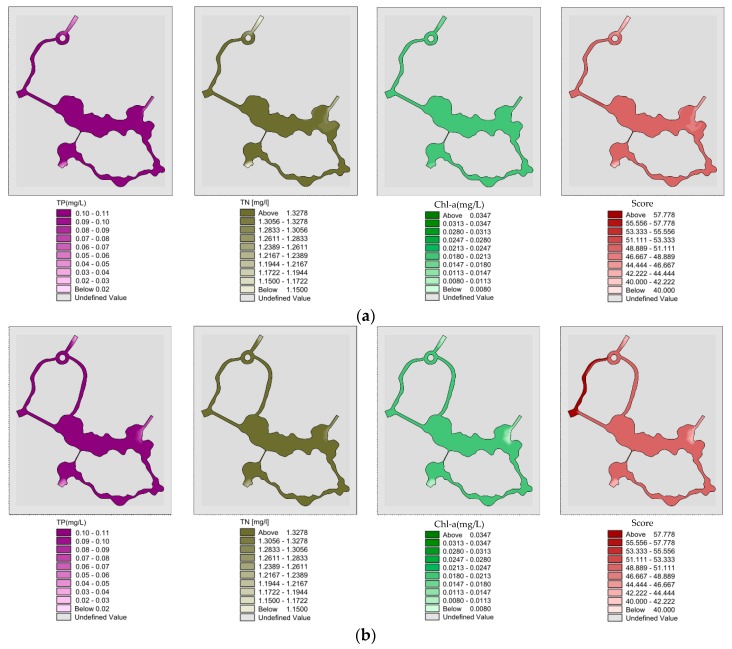
(**a**) Spatial distribution of “unilateral” river TP, TN, Chl-a and eutrophication scores without water diversion in winter; (**b**) Spatial distribution of “bilateral” river TP, TN, Chl-a and eutrophication comprehensive scores in winter without water diversion.

Spatial distribution of “unilateral” river TP, TN, Chl-a and eutrophication scores without water diversion in winter (Figure 18a). The mean values of TP, TN, Chl-a and eutrophication score of the wading area after the “unilateral” channel model reached the completely steady state were 0.103, 1.36, and 0.026 mg/L and 51.6. Spatial distribution of “bilateral” river TP, TN, Chl-a and eutrophication comprehensive scores in winter without water diversion (Figure 18b). The mean values of TP, TN, Chl-a and eutrophication score of the wading area after the “bilateral” channel model reached the completely steady state were 0.101, 1.355 and 0.02 mg/L and 50.6, respectively.

#### 3.2.5. Analysis of Water Quality and Eutrophication with Pumping Diversion in Winter

**Figure 19:**
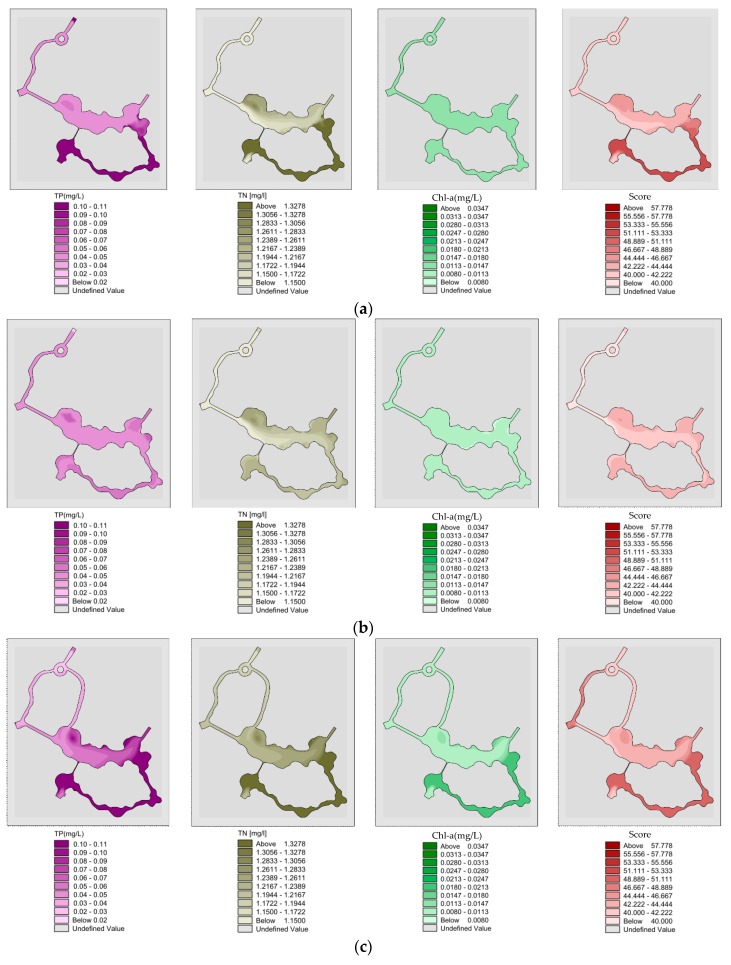

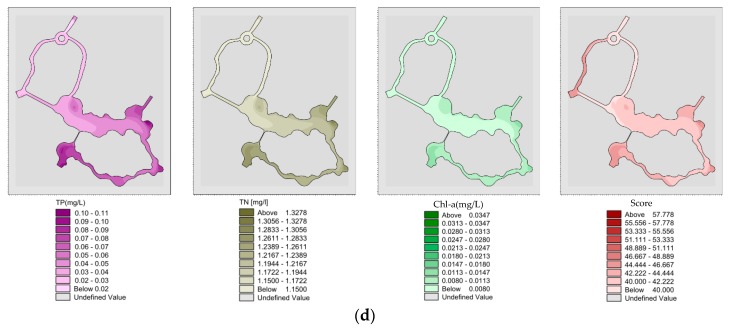
(**a**) Spatial distribution of “unilateral” river TP, TN, Chl-a and eutrophication scores after pumping 22 h in winter, when river model reached a completely steady state; (**b**) Spatial distribution of “unilateral” river TP, TN, Chl-a and eutrophication scores after pumping for 30 h in winter; (**c**) Spatial distribution of “bilateral” rivers TP, TN, Chl-a and eutrophication score after pumping. 22 h in winter; (**d**) Spatial distribution of “bilateral” rivers TP, TN, Chl-a and eutrophication score after pumping for 30 h in winter.

Spatial distribution of “unilateral” river TP, TN, Chl-a and eutrophication scores after pumping 22 h in winter, when river model reached a completely steady state (Figure 19a). The wading TP, TN, Chl-a and eutrophication comprehensive scores were 0.084, 1.279 and 0.014 mg/L and 46.2,; Spatial distribution of “unilateral” river TP, TN, Chl-a and eutrophication scores after pumping for 30 h in winter (Figure 19b). The model reached a completely steady state with mean TP, TN, Chl-a and eutrophication score values in the wading area of 0.049, 1.197 and 0.009 mg/L and 42.3 respectively; Spatial distribution of “bilateral” rivers TP, TN, Chl-a and eutrophication score after pumping. 22 h in winter (Figure 19c). After pumping for 22 h, model reaches a completely steady state. The mean values of TP, TN, Chl-a and eutrophication score in wading area were 0.082 mg/L, 1.277 mg/L, 0.013 mg/L and 45.7 respectively; Spatial distribution of “bilateral” rivers TP, TN, Chl-a and eutrophication score after pumping for 30 h in winter (Figure 19d). After 30 h of pumping, the “bilateral” rivers model reached a completely steady state. The mean values of TP, TN, Chl-a and eutrophication score in wading area were 0.047 mg/L, 1.195 mg/L, 0.008 mg/L and 41.8 respectively.

**Figure 20:**
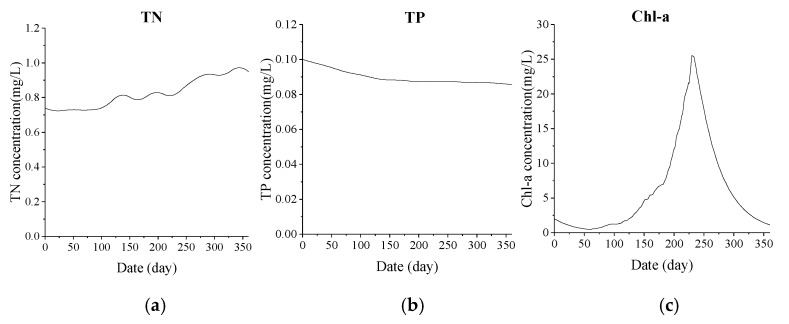
Predicted value of TN, TP, Chl-a concentration throughout whole year. (**a**) TN concentration; (**b**) TP concentration; (**c**) Chl-a concentration.

The annual variation of TN, TP and Chl-a concentration of water body calculated from the established eutrophication model is shown in Figure 20.

Based on the TN, TP and Chl-a predicted concentrations calculated by the model, the assessment is made according to the current domestic lake eutrophication score and classification criteria as stipulated in the “Assessment Methods of Surface Water Environmental Quality”. The monthly eutrophication scores are given in Table 7. The table shows that throughout the year there was mild eutrophication in June to October, of which the most serious occurred in August.

From the water quality and eutrophication model space and time prediction shows:

(1) In the layout of the “unilateral river” and “bilateral rivers” schemes, TP and TN meet the requirements of landscape and recreation with the water diversion, and the overall water quality meets the requirements. Spring and summer correspond to the tourist season and the light temperature is appropriate, the degree of eutrophication heavier, while in winter it is lighter; (2) In the absence of water diversion in summer, due to the slow flow rate in the wading area, the entire wading area under the “unilateral river” and “bilateral rivers” layout is in a mild eutrophication status, in particular in the southeast corner of the Shuijie 1–2 corner, due to dense passenger flow, resulting in heavier human point source pollution. As the winter temperature is low and the light intensity is low and short, is not conducive to algae growth, chlorophyll a concentration is low, and there is less eutrophication. (3) In the condition of pumping, the water quality and eutrophication in the location where #1 push pump is located is better than that of “bilateral” rivers, because “unilateral” river lacks the Shuijie Section 3 and more water flows through Shuijie Section 1 and Section 2. (4) In the case of sluice diversion, the water quality and eutrophication in the west side of center landscape lake area the “unilateral” river was inferior to the “bilateral” river because of poor water flow. (5) In overview of the eutrophication occurred region, for the use of three kinds of water diversion: sluice diversion, pumping and push pump, sluice diversion and pumping improved the entire wading area as a whole. For the commercial area of Shuijie where cyanobacteria are prone to occur, as well as the central landscape lake and the southeast corner of the wading area, push pump or algae salvaging are provided for local water quality improvement.

### 3.3. Comprehensive Benefit Analysis

According to the comprehensive benefit analysis of “unilateral” and “bilateral” rivers from four aspects of comprehensive project cost, tourism benefit, environmental benefit and river layout, we can see: (1) From the analysis of environmental benefits: there is not much difference between the two river layouts. (2) From the economic and social benefits analysis: from the short-term benefits point of view, we recommended “unilateral” river. From a long-term benefit point of view, “bilateral” rivers will have more domestic and international market share of the source, so that both social benefits and economic benefits. See Table 8 for details.

### 3.4. Overall Scheduling Scheme

The Neijiang lake normal water level is 3.9 m, the minimum control level when changing water is 3.6 m, the highest control level is 4.1 m, the highest flood control water level is 5.9 m. The internal water system and Neijiang lake water levels are basically the same, and the designed playground water level is between 3.60~4.10 m. In the rainy season, once the water level in the playground area exceeds 4.0 m, the pumping station will start to drain water. When the water level in the river reaches 3.8 m, the pumping station will be stopped. Based on the comparison and selection of programs, the final overall scheduling solution is determined. Flood control and drainage scheduling are implemented to ensure that floods do not occur in the wading area. When the approach channel is opened, the sluice will start water diversion and the pump station will start diversion when it cannot be opened. According to the temperature of the corresponding scheduling days, we control the pump diversion days. When the temperature is higher than 15 °C and no water diversion using push pump to speed up the retention, the flow area slows. The overall schedule is shown in Table 9.

Priority 1: prioritize flood control and drainage schedule to ensure that no disaster occurs in the wading area. The specific drainage program is as follows: (1) storms when the water level over 4.0 m, close three sluices and one culvert, open 2 sets of pumps for drainage. (2) when the water level is lower than 3.9 m, stop one pump (when the water level exceeds 3.9, all will resume). (3) when the water level is lower than 3.8 m stop all pumps.

Priority 2: when the playground water level is low(generally less than 3.3 m), affecting the operation of the water project, shut down the three sluices and one culvert and open the 2 pumps of #1 to divert water to stop at 4.1 m, or by sluice diversion hold the wading area operating water level.

Priority 3: when the tidal level of the Yangtze River is higher than the water level of Neijiang Lake (3.9~4.1 m), the approach channel will open its sluice and divert water to open the three sluices and one culvert to change the playground water.

Priority 4: when there is no sluice diversion use pumping: according to the temperature of the corresponding scheduling days, open the #1 pump diversion. When the temperature is higher than 15 °C and no water diversion when the push pump is used to speed up the retention, slow the flow area.

## 4. Conclusions

The purpose of this article was to regulate the water environment of the artificial Playground Lake-interconnected Yangtze River system. Playground location choice, construction, mathematical modelling, water quality forecasting and other aspects were analyzed, and after a comprehensive benefit analysis a scheduling scheme was proposed. The eutrophication model presented in the paper was used to simulate under different water diversion scenarios. According to the simulation and numerical analysis, the main conclusions are as follows:

(1) The soil and groundwater pollutants were analyzed for the site selection and the effects of pollutants in the chemical plant, domestic waste landfill and shipyard were known. According to the excavation scope of the playground, the impermeable range of the artificial lake is obtained, and different types of slope protection (scenery stone revetment, soil slope protection, wood pile slope protection) are confirmed in combination with the landscape. The pollution source during the operation of playground is predicted by the Tourist Activities Disturbance Index method.
(2) In order to ensure that the water quality and eutrophication of the original planning river meet the water requirements for landscape entertainment, three water diversion schemes (pumping, sluice, push pump) are proposed to control the eutrophication of the water in the wading area. In order to improve the flow field in the wading area, three new river programs (“roughed” river, “bilateral” river, “unilateral” river) are proposed. Due to the complexity of the “roughed” river engineering, two river layout schemes, “bilateral” and “unilateral”, are recommended.
(3) In the layout of the “unilateral river” and “bilateral rivers” schemes, TP and TN meet the requirements of landscape and recreation by the water diversion, and the overall water quality meets the requirements. Spring and summer correspond to the tourist season and the light temperature is appropriate, the degree of eutrophication heavier, while in winter it is lighter. In the absence of water diversion during summer, the entire wading area under the “unilateral” and “bilateral” rivers is under mild eutrophication. In winter, due to the low temperature, light intensity and shorter duration, conditions are not conducive to the growth of algae, the concentration of chlorophyll a is low, and the degree of eutrophication is lighter. In the condition of pumping, the water quality and eutrophication in the location where #1 push pump is located is better than that of “bilateral” rivers. In the case of sluice diversion, the water quality and eutrophication in the west side of center landscape lake area the “unilateral” river was inferior to the “bilateral” river because of poor water flow. In view of the eutrophication area, three types of water transfer methods were used: sluice diversion, pump diversion, and push pump cycle. sluice and pump diversion leading to an overall improvement over the entire wading area, while push pump provided partial improvements.
(4) Comparing the comprehensive schemes, we determine the final overall scheduling schemes: Priority 1: prioritize flood control and drainage schedule to ensure that no disaster occurs in the wading area. The specific drainage program is as follows: ① storms when the water level over 4.0 m, close three sluices and one culvert, open 2 sets of pumps for drainage. ② when the water level is lower than 3.9 m, stop one pump (when the water level exceeds 3.9, it will resume pumping). ③ when the water level is lower than 3.8 m stop all pumps. Priority 2: when playground water level is low, affecting the operation of the water project (generally less than 3.3 m), shut down the three sluices and one culvert and open the 2 pumps of #1 to divert water to stop at 4.1 m, or by sluice diversion holding the wading area operating water level. Priority 3: when the tidal level of the Yangtze River is higher than the water level of Neijiang (3.9~4.1 m), the approach channel will open its sluice and divert water to open the three sluices and one culvert to change the playground water. Priority 4: when there is no sluice diversion use pumping: according to the temperature of the corresponding scheduling days, open the #1 pump diversion. When the temperature is higher than 15 °C and no water diversion when the use of push pump to speed up the retention, and slow the flow area.

## Figures and Tables

**Table 1:** Main areas of the playground pollutant concentration (mg/L).

Entertainment Activities	TN	TP	COD
Attraction vendors	0.63	0.16	2.84
Water Bus	1.96	0.25	3.09
Ocean World	1.14	0.19	2.84
Polar Dry Skiing	1.54	0.26	2.41
Shuijie Business District	1.89	0.23	2.94
WC	0.53	0.08	0.68

**Table 2:** Main water quality and eutrophication parameters.

Number	Parameters	Value [35,36,37,38]	Unit
1	Chl-a growth rate	1.8	per day
2	Chl-a sedimentation rate	0.11	per day
3	Sediment oxygen consumption	0.5	per day
4	Nitrifying oxygen demand of ammonia nitrogen	3.42	g O_2_/g NH_4_-N
5	Nitrite denitrification oxygen demand	1.14	g O_2_/g NO_2_-N
6	Denitrification rate	0.1	per day
7	Phosphate degradation rate	0.06	g P/m^3^/day

**Table 3:** Specification parameters of “three sluices and one culvert”.

Number		Size
#1	sluice	Net width of 9 m, bottom elevation of 1.2 m
	pump	A single pump flow of 1.85 m^3^/s with a total of two
#2 sluice		Net width of 10 m, sluice bottom elevation of 1.0 m
#3 sluice		Net width of 9 m, sluice bottom elevation of 1.5 m
#4 culvert		Net size of 2 m × 2 m

**Table 4:** “Three sluices and one culvert” scheduling plan during pump diversion.

Time (24 h)	Pump	#1 Sluice	#2 Sluice	#3 Sluice	#4 Sluice	Remarks
Before pumping	Close	Open	Open	Open	Open	/
0:00~22:00	Open	Close	Open	Close	Close	Shuijie and central landscape lake area water diversion
22:00~6:00^+1^	Open	Close	Close	Open	Open	All areas water diversion
6:00^+1^~7:00^+1^	Close	Close	Open	Open	Open	Close #1 sluice for 1 h to prevent backwater
After pumping	Close	Open	Open	Open	Open	Open the push pump

Note: time^+1^ means the next day.

**Table 5:** Push pump turn on and off schedule.

Schedule	Temperature	Turn on and Off
1	<15 °C	Off
2	>15 °C	Off at sluice diversion
		Off at #1 pump diversion
		Turn on the rest time

**Table 6:** Model calculation scheme.

Program	Wind Direction	Temperature	Water Diversion
1	SE	28 °C	No
2			Pump
3			Sluice
4	NE	7 °C	No
5			Pump

**Table 7:** Predicted results of eutrophication.

Month	Playground Lake	
	Score	Evaluation Results
1	42.74	Moderate eutrophication
2	39.19	Moderate eutrophication
3	40.00	Moderate eutrophication
4	42.99	Moderate eutrophication
5	46.95	Moderate eutrophication
6	50.16	Mildly eutrophication
7	53.33	Mildly eutrophication
8	56.69	Mildly eutrophication
9	55.74	Mildly eutrophication
10	52.71	Mildly eutrophication
11	49.46	Moderate eutrophication
12	46.23	Moderate eutrophication

**Table 8:** Comparison of comprehensive benefits of “unilateral” and “bilateral” rivers.

Category		“Unilateral” River	“Bilateral” Rivers	Recommendation
Project costs	Construction	165,000 yuan	20.16 million yuan	“Unilateral” river
	Operating	116,000 yuan/a	100,000 yuan/a	
Travel profit		330 million yuan	340 million yuan	“Bilateral” river
Environmental benefits		West Lake landscape center stay, easy to produce cyanobacteria	#1 Push pump position to improve the water diversion effect is not obvious	After dispatching engineering measures, the environmental benefits of the two are no much difference
River layout		“Bilateral” rivers-point and line combination of a complete “garden-style” playground		“Bilateral” river

**Table 9:** Overall scheduling scheme table.

Priority 1	Priority 2	Priority 3	Priority 4		
Drainage Program	Operational Assurance Program	Ecological Diversion Program			
	Sluice or Pump Diversion	Sluice Diversion	Pump Diversion		
In rainy season, when the rainfall level is higher than 4.0 m.	During the project operation period, the river water level is lower than 3.3.	Open the sluice in the diversion channel Note: Sluice diversion is generally carried out in June, July and August (temperature above 23 °C), twice per month.	Water temperature	#1 pump diversion	Push pump
			<15 °C	15 d/time, each time 30 h	No
			15 °C–25 °C	10 d/time, each time 30 h	No pumping and no sluice diversion
			>25 °C	7 d/time, each time 30 h	No pumping and no sluice diversion

## Appendix A

**Table A1:** Initialization of Parameters.

**Parameter**	**Value**
Water level	2.67 m
Temperature	28 °C
Flow velocity	0 m/s
COD	3.03 mg/L
Ammonia nitrogen	0.15 mg/L
Total phosphorus	0.039 mg/L
Total nitrogen	1.182 mg/L
Chl-a	0.016 mg/L
